# Risk stratification tools for branch‐duct intraductal papillary mucinous neoplasms of the pancreas

**DOI:** 10.1002/ueg2.12205

**Published:** 2022-02-05

**Authors:** Alberto Balduzzi, Roberto Salvia, Matthias Löhr

**Affiliations:** ^1^ Department of Surgery, Dentistry, Paediatrics and Gynaecology Unit of General and Pancreatic Surgery The Pancreas Institute Verona University of Verona Verona Italy; ^2^ Department for Digestive Diseases Karolinska University Hospital Stockholm Sweden

**Keywords:** acute pancreatitis, oncology, pancreas, pancreatic cancer, surgery

Branch duct papillary mucinous neoplasms (BD‐IPMNs) of the pancreas represent the majority of pancreatic cystic neoplasms (PCNs) diagnosed during routine radiologic examinations and are deemed to have the least likelihood of harboring or developing malignancy.^1^ While the true risk of progression and malignant degeneration of presumed BD‐IPMN is yet to be untangled, thanks to international scientific efforts,^2^ current guidelines^3^, ^4^, ^5^ advise for surveillance also for small pancreatic cysts to detect any signs of progression to malignancy and possibly assessing the best time for surgery, notwithstanding the significant burden on patients and healthcare resources.^6^, ^7^ Indeed, patient selection is crucial both to avoid unnecessary surgery and to recommend surveillance if the risk of disease progression cannot be ruled out. Prediction models have been built to improve the correct identification of high‐risk IPMNs and thus the selection of patients for surgery.^8^, ^9^, ^10^ However, there are currently no tools to recommend the best IPMN surveillance strategy and to distinguish those who might not warrant surveillance at all.^7^

In a recently published article, Overbeek et al.^11^ overturn the classical model and present a stratification tool aimed to identify patients at the lowest risk of developing worrisome features or high‐risk stigmata. The model was developed and validated between three International high‐volume centers. The Dutch–American Risk stratification Tool (DART‐1) is a negative prediction tool aimed to identify those BD‐IPMNs requiring less intense surveillance. The authors are certainly to be commended for their work. Whilst perceived positively by physicians, surveillance can have downsides for the patient and healthcare resources.^12^ Therefore, we support the endeavor to improve patient selection and identify those who are at low risk of progression, even more during the current Covid‐19 pandemic. However, it is questionable whether the currently available scientific evidence is sufficient to support these findings. Indeed, we will need large international observational series, with long‐time surveillance. The DART‐1 was built on guidelines published in 2012^13^ and will certainly benefit from a refinement centered on the updated 2017 and 2018 guidelines.^3^, ^4^ As an exponential increase in publications focused on IPMNs has occurred in recent years, these latter guidelines would also benefit from updating, which is currently in progress.

Recently, Mestier et al.^14^ published an interesting patient‐level meta‐analysis aiming to evaluate the appropriateness of surgical management in high‐risk individuals (HRI), defined as the presence of morphological abnormalities suggesting the development of pancreatic cancer during surveillance. Appropriateness was confirmed in less than half of HRI, advising that patient selection should be optimized. Moreover, the importance of the proper timing for surgery is underlined by the increased survival rate of patients in case of high‐grade dysplasia compared to invasive cancer (2‐year rate of 100% vs. 55.8%), albeit acknowledging the limitation of the short follow‐up period after surgery. The study does not provide any information regarding the natural history of pancreatic pre‐malignant lesions such as BD‐IPMN in HRIs, and the nomogram build to predict the appropriateness of prophylactic pancreatic surgery in HRI following screening appears to be too broad to be routinely applied.^14^

An inspiring shared‐decision program has been designed by Scholten et al.,^15^ focusing on total pancreatectomy for HRIs to develop pancreatic malignancies. What really stands out from this study is the importance of engaging the patient in the decision‐making process. Not only must the consensus be present among different specialists, but it must also be shared and understood in its risks and benefits by the patient himself. Therefore, if a BD‐IPMN is suspected, the physician is required to explain to the patient the risk of surveillance, the possible psychological burden, the need for radiological examinations, the change in progression, and the possibility to shift the management to surgery with its associated risks. Likewise, the patient should be appraised that the indication for life‐long follow‐up persists even after surgery because of the risk of recurrence or new cancer in the remaining pancreas.

In conclusion, it is all about balance, and balance itself cannot only be referred to as a precise mathematical equation discounting both clinician and patient insights. Future studies should focus on large observational series of presumed BD‐IPMN without signs of degeneration, aiming to detect the true radiological and biological dynamic predictors associated with BD‐IPMN progression to malignancy. In fact, we cannot rely on a single frame to predict the possible evolution of the BD‐IPMN. To date, it does not seem advisable to recommend stratification tools or nomograms inevitably built on the limitations of currently available literature. Awaiting to receive the results of ongoing studies, according to recent evidence, we can speculate that the answer lies in a combination of variables including radiologic and endoscopic examinations, cystic fluid analysis, the willingness of the patient, and the intuition of the physician. Proving it through collaborative international studies will represent the real breakthrough in IPMNs management.

## CONFLICT OF INTEREST

The authors have no conflicts of interest to declare.

## Data Availability

Data sharing is not applicable to this article as no new data were created or analyzed in this study.
